# Can prone positioning be a safe procedure in patients with acute brain injury and moderate-to-severe acute respiratory distress syndrome?

**DOI:** 10.1186/s13054-020-03454-9

**Published:** 2021-01-18

**Authors:** Pauline Bernon, Ségolène Mrozek, Guillaume Dupont, Frédéric Dailler, Anne-Claire Lukaszewicz, Baptiste Balança

**Affiliations:** 1 Hospices Civils de Lyon, Hôpital Pierre Wertheimer, Département d’anesthésie réanimation, Hôpital Neurologique Pierre Wertheimer, 59 Boulevard Pinel, 69500 Bron, Lyon, France; 2 Département d’anesthésie Réanimation, Centre Hospitalier Universitaire de Toulouse, Université Toulouse 3-Paul Sabatier, Toulouse, France; 3 Département d’anesthésie Réanimation, Centre Hospitalier Universitaire de Saint-Etienne, Saint-Priest-en-Jarez, France; 4 EA 7426 PI3 (Pathophysiology of Injury-Induced Immunosuppression), Hospices Civils de Lyon/Université Claude Bernard Lyon 1/bioMérieux, Hôpital E. Herriot, 5, place d’Arsonval, 69437 Lyon Cedex 03, France; 5 Lyon Neuroscience Research Center, U1028, UMR 5292, Bron, France

Dear Editor,

One third of patients with severe brain injury develop lung complication that affect their prognosis. Prone positioning (PP) improves the outcome of patients with an acute respiratory distress syndrome (ARDS) [1], but its effect on patients with acute brain injury is still debated. While it improves oxygenation, the impact of PP on intracranial pressure (ICP) remains controversial: PP has been reported not to affect ICP [2] and conversely to increase ICP, thereby worsening brain injuries [3]. There is currently no consensus on criteria to identify patients who will safely benefit from PP [4]. Therefore, the aim of the present study was to evaluate the safety and efficacy of PP in patients with acute brain injury and moderate-to-severe ARDS.

A retrospective analysis in three French intensive care units was conducted. A query on digital medical records identified 27 patients with an ICP, moderate-to-severe ARDS (according to the Berlin criteria) and PP. Data were collected before and during the first PP. Patients who had at least one ICP measurement > 25 mmHg were considered as having intracranial hypertension (IH) as it is associated with a poor prognosis and used to consider a decompressive craniectomy [5].

A total of 10 (37.0%) patients had traumatic brain injuries, 11 (40.7%) subarachnoid haemorrhage, and 7 (25.9%) haemorrhagic stroke (Table1). During PP, the median [IQR] PaO_2_/FiO_2_ increased significantly from 100 [89–126] before to 216 [171–257] after PP (Wilcoxon test, *p* < 0.001) and remained significantly higher back to supine position (146 [122–186], Wilcoxon test, *p* = 0.002). IH occurred in 14 (51.8%) patients. They had a significantly higher median [IQR] ICP before PP onset (20 [13–26] mmHg) compared to patients without IH (11 [7–12] mmHg, Mann–Whitney test, p = 0.005) and a greater ICP increase during PP (+ 19 mmHg [13–20] vs + 6 mmHg [3–8], Mann–Whitney test, *p* = 0.025), suggestive of a poorer brain compliance. PP was discontinued due to a sustain ICP increase in 5 patients (Fig. 1a).

**Table 1:** Population characteristics

	Total population (n = 27)	IH (n = 14)	No-IH (n = 13)	p*
Age, median [IQR]	46 [36–55]	46 [36–50]	46 [37–56]	0.981
Female, n (%)	5 (18.5%)	2 (14.3%)	3 (23.1%)	0.648
BMI, median [IQR]	26 [23–31]	26 [22–30]	26 [23–33]	0.601
Traumatic brain injury, n (%)	10 (37.0%)	6 (42.9%)	4 (30.8%)	0.694
Subarachnoid haemorrhage, n (%)	11 (40.7%)	5 (35.7%)	6 (46.1%)	0.703
Haemorrhagic stroke, n (%)	7 (25.9%)	4 (28.6%)	3 (23.1%)	1.000
Ischemic stroke, n (%)	1 (3.7%)	0 (0%)	1 (7.7%)	0.481
Aspiration pneumonia, n (%)	13 (48.1%)	7 (50.0%)	6 (46.1%)	1.000
Ventilator-associated pneumonia, n (%)	14 (51.8%)	7 (50.0%)	7 (53.8%)	1.000
Severity				
SAPSII, median [IQR]	42 [34–53]	47 [41–55]	39 [30–46]	0.076
Glasgow Coma Scale at intubation, median [IQR]	6 [4–8]	6 [4–8]	6 [4–7]	0.769
First ICP measure, median [IQR]	22 [12–29]	24 [19–37]	16 [8–26]	0.274
IH treatment				
Craniectomy, n (%)	3 (11.1%)	1 (7.1%)	2 (15.4%)	0.595
Hypothermia, n (%)	5 (18.5%)	3 (21.4%)	2 (15.4%)	1.000
Osmotherapy, n (%)	11 (40.7%)	7 (50,0%)	4 (30.8%)	0.440
Thiopental administration, n (%)	10 (37.0%)	6 (42.9%)	4 (30.8%)	0.694
At least one of IH treatment, n (%)	14 (51.8%)	8 (57.1%)	6 (46.1%)	0.706
EVD, n (%)	14 (51.8%)	7 (50.0%)	7 (53.9%)	1.000
ARDS treatment				
Neuromuscular blockade, n (%)	27 (100%)	14 (100%)	13 (100%)	
PP number, median [IQR]	1 [1–2]	1 [1–2]	1 [1–3]	0.295
Duration of PP (hours), median [IQR]	14 [9–19]	13 [8–17]	16 [11–20]	0.305
First PP delay, median days [IQR]	5 [4–7]	6 [5–7]	5 [4–6]	0.279
Tidal volume mL/kg, median [IQR]	6.8 [6.4–7.5]	6.7 [6.4–7.5]	6.9 [6.4–7.5]	0.843
Parameters before PP				
Initial ICP (mmHg), median [IQR]	13 [8–20]	20 [13–26]	11 [7–12]	0.005
Initial CPP (mmHg), median [IQR]	75 [66–82]	67 [64–75]	79 [77–87]	0.041
Initial PEEP (cmH_2_0), median [IQR]	10 [9–12]	10 [9–11]	10 [9–12]	0.657
Initial FiO_2_, median (%) [IQR]	80 [60–89]	80 [71–100]	67 [60–81]	0.231
Initial plateau pressure (cmH_2_0), median [IQR]	23 [21–27]	23 [21–29]	23 [21–26]	0.689
Initial PaO_2_/FiO_2_, median [IQR]	100 [89–126]	99 [88–113]	109 [93–142]	0.481
Initial PaO_2_ (mmHg), median [IQR]	78 [74–95]	78 [74–90]	77 [74–99]	0.903
Initial PaCO_2_ (mmHg), median [IQR]	43 [37–47]	43 [38–46]	44 [36–48]	0.884
Outcome				
Mechanical ventilation duration (days), median [IQR]	23 [11–36]	22 [7–35]	23 [16–37]	0.395
Modified Rankin Scale at ICU discharge, median [IQR]	4 [4–5]	4 [4–6]	4 [4–5]	0.853
Mortality, n (%)	7 (25.9%)	4 (28.6%)	3 (23.1%)	1.000

*BMI* Body mass index, *CPP* cerebral perfusion pressure, *EVD* external ventricular drainage, *FiO*_*2*_ inspiratory fraction of oxygen, *ICP* intracranial pressure, *ICU* intensive care unit, *IH* intracranial hypertension, *PEEP* positive end-expiratory pressure, *PP* prone positioning, *SAPSII* Simplified Acute Physiology Score 2

*p value IH group versus no-IH group (using the Mann–Whitney or the Fisher’s test)

**Fig. 1:**
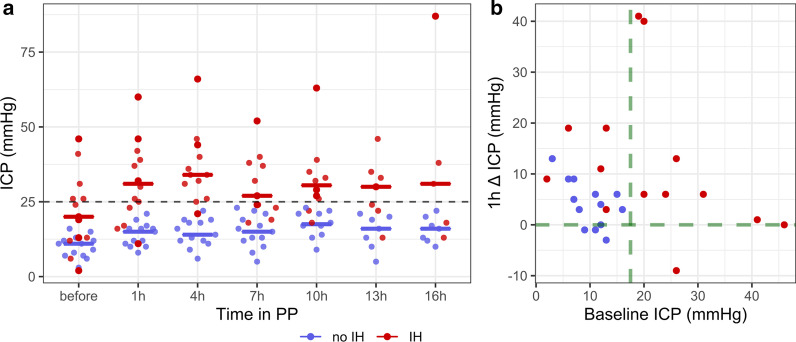
Intracranial pressure changes during prone positioning. Each point represents a patient, in blue patients who did not experience intracranial hypertension (IH) during prone positioning (PP) and in red patients who did. **a** ICP changes over the PP session; solid lines indicate the median intracranial pressure (ICP) values of each group. **b** ICP changes 1 h after the PP onset according to the initial ICP before PP; when the initial ICP was below 17.5 mmHg and the ICP did not increase PP took place without IH (green lines), whereas an ICP above 17.5 mmHg or an ICP elevation over 10 mmHg were predictive of IH

All patients with an ICP > 17.5 mmHg prior to PP had an IH. Among patients with an ICP < 17.5 mmHg before PP onset, 13/18 (72%) had a safe PP session without IH. Rather than a single threshold of ICP changes, a grey zone approach was used to predict (i.e. sensitivity, Se > 90%) or exclude (i.e. specificity, Sp > 90%) a safe PP. The absence of ICP increase 1 h after the PP onset is suggestive of a preserved brain compliance and predicted a safe procedure (Sp = 93%), while an ICP elevation > 10 mmHg predicted the occurrence of IH (Sp = 93%). When the initial ICP was < 17.5 mmHg and did not increase 1 h after PP onset the manoeuvre took place without IH (Fig. 1b). Brain oxygen partial pressure was available for 4 patients and rose from 20.5 [18.8–23.5] mmHg to 28 [22–31] mmHg during PP.

The main limitations of this study are due to its retrospective design. The modalities for performing manually PP were not available although it can influence its tolerance [6]. In addition, the data collected during PP from the ICU software were sampled hourly at a specific time and may not reflect the average of the hour. Only 4 patients had an intracranial oxygenation probe improved during PP and suggested a preserved cerebral blood flow despite the ICP increase. Finally, the management of IH was not subject to protocol.

To conclude, we would argue for assessing the brain compliance before PP (e.g. transcranial Doppler), ICP, and the tolerance to an obstacle to venous return. Moreover, ICP changes within 1 h after PP onset could be useful to choose to pursue PP or not, as well as cerebral multimodal monitoring to evaluate PP tolerance. This strategy needs to be evaluated in a prospective clinical trial.

## Data Availability

The datasets used during the current study are available from the corresponding author on reasonable request.
